# Suppression of Inflammation-Associated Kidney Damage Post-Transplant Using the New PrC-210 Free Radical Scavenger in Rats

**DOI:** 10.3390/biom11071054

**Published:** 2021-07-19

**Authors:** Torsten R. Goesch, Nancy A. Wilson, Weifeng Zeng, Bret M. Verhoven, Weixiong Zhong, Maya M. Coumbe Gitter, William E. Fahl

**Affiliations:** 1 Obvia Pharmaceuticals Ltd., Madison, WI 53719, USA; torsten@goesch.com; 2 Department of Surgery, Division of Organ Transplant, University of Wisconsin-Madison, Madison, WI 53706, USA; nawilson@medicine.wisc.edu (N.A.W.); wzeng28@wisc.edu (W.Z.); verhoven@surgery.wisc.edu (B.M.V.); wzhong3@wisc.edu (W.Z.); 3 Department of Oncology, Wisconsin Institutes for Medical Research, University of Wisconsin-Madison, Madison, WI 53706, USA; coumbe@wisc.edu

**Keywords:** kidney allograft, kidney rejection, ischemia

## Abstract

Allograft kidney transplantation, which triggers host cellular- and antibody-mediated rejection of the kidney, is a major contributor to kidney damage during transplant. Here, we asked whether PrC-210 would suppress damage seen in allograft kidney transplant. Brown Norway (BN) rat kidneys were perfused in situ (UW Solution) with or without added 30 mM PrC-210, and then immediately transplanted into Lewis (LEW) rats. 20 h later, the transplanted BN kidneys and LEW rat plasma were analyzed. Kidney histology, and kidney/serum levels of several inflammation-associated cytokines, were measured to assess mismatch-related kidney pathology, and PrC-210 protective efficacy. Twenty hours after the allograft transplants: (i) significant histologic kidney tubule damage and mononuclear inflammatory cell infiltration were seen in allograft kidneys; (ii) kidney function metrics (creatinine and BUN) were significantly elevated; (iii) significant changes in key cytokines, i.e., TIMP-1, TNF-alpha and MIP-3A/CCL20, and kidney activated caspase levels were seen. In PrC-210-treated kidneys and recipient rats, (i) kidney histologic damage (Banff Scores) and mononuclear infiltration were reduced to untreated background levels; (ii) creatinine and BUN were significantly reduced; and (iii) activated caspase and cytokine changes were significantly reduced, some to background. In conclusion, the results suggest that PrC-210 could provide broadly applicable organ protection for many allograft transplantation conditions; it could protect transplanted kidneys during and after all stages of the transplantation process—from organ donation, through transportation, re-implantation and the post-operative inflammation—to minimize acute and chronic rejection.

## 1. Introduction

End-stage renal failure causes greater than 1.2 million deaths annually worldwide [1]. Kidney transplantation is the preferred treatment for patients with end-stage renal disease. Over 90,000 kidney transplants are performed each year worldwide.

The transplant process, itself, induces significant cellular and organ injury to the kidney, which reduces long-term survival of the organ. The three primary insults to a kidney during an allograft transplant are (i) reactive oxygen and nitrogen species (ROS and RNS)-induced damage during cold ischemia (‘cold-storage’) [2], (ii) ROS-induced damage upon implant (‘re-perfusion injury’) [3], and (iii) post-allograft-transplant inflammation, which triggers the innate immune response and antibody-mediated rejection (ABMR) [4].

Neutrophils and macrophages migrate into the damaged transplant within 6 h of reperfusion and stimulate chemokine synthesis in resident dendritic cells that then activate T lymphocytes and recruit adaptive immune cells. Once these immune cells infiltrate the proximal tubule epithelial cells, they produce myeloperoxidase in neutrophils and nicotinamide adenine dinucleotide phosphate (NADPH) oxidase in macrophages, both of which contribute to local free radical production. These inflammatory processes lead to an activation of the complement pathway and further cell remodeling and lysis in the kidney allograft [5].

ABMR can occur as a result of either, or both, preformed alloantibody against the graft or through the *de novo* development of donor-specific antibody (dnDSA) [5,6,7]. The acute (min/days), transitioning to chronic (days/weeks), inflammatory response within the allograft kidney, with continuous production of ROS and inflammatory cytokines, can establish a severe, self-perpetuating response that causes kidney organ failure.

To better understand cellular and molecular pathways involved in the pathogenesis of kidney allograft inflammation and rejection, we developed and characterized a rat model that replicates most of the clinical criteria of innate immune response, ABMR and kidney organ loss [8]. This model has been used to evaluate a number of novel post-allograft transplant strategies.

The two currently acknowledged approaches for reducing the acute and long-term immune response against the kidney allograft are: (i) to increase the chance of finding a cross-matched donor, and (ii) to remove preexisting antibodies against the kidney allograft using desensitization protocols [9,10].

In the work described in this manuscript, we asked whether a third approach to suppress acute and longer-term inflammation severity would be beneficial. We administered the immediate-acting, free radical scavenger, PrC-210, both to the implanted allograft kidney and to the recipient rat, to determine whether inflammation-associated ROS damage could be suppressed. Though the concept of suppressing inflammation-associated ROS in kidney transplant is not new, the use here of the new, immediate-acting PrC-210 ROS scavenger is. Both immediate and chronic scavenging and inactivation of inflammation-generating, and generated, free radicals within the newly transplanted allograft kidney would significantly enhance the existing strategies to suppress allograft rejection and would provide another pathway to reduce post-transplant kidney cell damage, and with it, suppress Delayed Graft Function to improve survival of the kidney allograft.

PrC-210 is a new small-molecule, aminothiol, free radical scavenger [11]; it has no measurable nausea/emesis nor hypotension side effects [12]. Unlike traditional antioxidants that act *indirectly* over hours to days via NrF-2 to activate expression of protective genes [13], PrC-210 *directly* scavenges ROS to confer 100% protection in seconds [11]. PrC-210 was the most potent of the 13 commonly studied “antioxidants” screened in an assay that scored the ability of molecules to prevent x-ray-induced damage to naked DNA; the majority of the tested “antioxidants” showed no protection [14,15]. In a related assay, addition of PrC-210 30 s before a 60 s pulse of ●OH to naked DNA provided complete protection against the ●OH insult that induced >95% DNA damage in unprotected controls [16]. In two previous rodent kidney transplant studies [16,17], PrC-210 was shown to suppress ROS-induced kidney damage induced during (i) 30 h cold storage [17] and (ii) reperfusion injury upon implant [16] to *background* levels, thus removing two substantial sources of injury to the transplanted kidneys. The PrC-210 molecule has also been shown to suppress free radical-induced injury in several other organ settings [15,18]. Thus, we hypothesized that PrC-210 should also be able to protect an allograft against oxidative stress that is generated by BOTH (i) cellular- and (ii) antibody-mediated rejection processes that produce free radicals as a byproduct.

To explore this hypothesis, we developed a new rat model which avoided the induction of major ischemic and reperfusion events, and administered PrC-210 both pre- and post-implantation. Brown rat kidneys were flushed with UW solution containing PrC-210 and immediately transplanted into syngeneic Lewis rat recipients. Cold ischemic time was virtually eliminated. Immediately following implant, and for 8 h following kidney implant, recipient rats received systemic PrC-210 injections at doses that would enable continuous free radical-scavenging within the transplanted kidney. Transplanted kidneys and blood plasma were then harvested 20 h following transplant to enable measurement of both PrC-210-conferred (i) suppression of inflammatory byproducts and (ii) kidney protection.

## 2. Materials and Methods

### 2.1. Animals

Adult (200–250 g) male Lewis and BN rats were purchased from Envigo (Indiannapolis, IN, USA) and housed in the animal care facility at the University of Wisconsin in Madison, WI, USA. All procedures were performed in accordance with the Animal Care and Use Policies at the University of Wisconsin. Animal health maintenance, including animal deaths, room temperature, 12 h light/dark cycles, and cage cleaning, among other sanitation duties, were performed daily by animal care staff. Food and water were available ad libitum. This research was prospectively approved by School of Medicine and Public Health Institutional Animal Care and Use Committee at the University of Wisconsin (Animal Protocol #M005204). All groups contained 4–6 animals.

### 2.2. Materials

Synthesis of the PrC-210 HCl aminothiol, a preclinical molecule, was described separately [19,20]. PrC-210 HCl crystals (3-(methylamino)-2-(methylaminomethyl)propane-1-thiol) are stored under a nitrogen atmosphere at −20 °C, and even with routine thawing, use, and re-storage, crystalline PrC-210 is completely stable for greater than 4 years by mass spectrometry analysis. Other chemical reagents were obtained from Sigma Aldrich (St. Louis, MO, USA). UW Organ Preservation Solution was purchased from Bridge to Life, Columbia, SC, USA.

### 2.3. Surgical and Experimental Procedure

**Figure 1:**
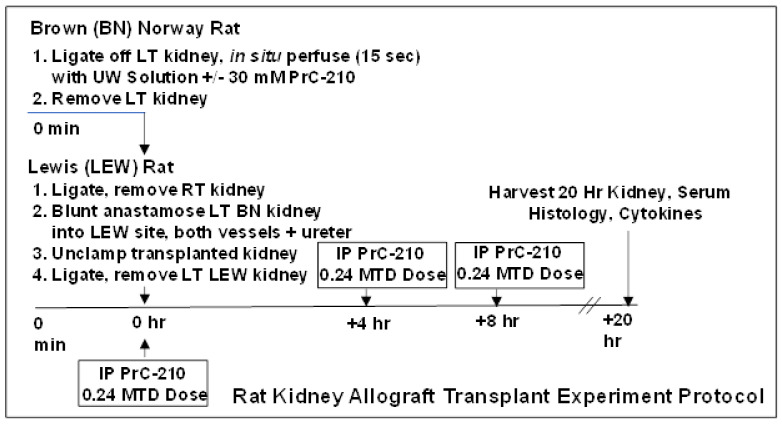
Experimental schematic showing surgery and PrC-210 administration times for the BN rat kidney transplants into LEW rats. LT (**left**), RT (**right**), MTD (maximum tolerated dose), and IP (intraperitoneal).

The transplant procedure used in these experiments is shown in Figure 1. In the BN donor rat, after double ligation of the aorta, ligation of the right renal artery and vein, and surgical section of the left renal vein, the left rat kidney was perfused in situ using 5 mL of room temperature UW Solution (over a 15 s period). The perfusate was either UW Solution alone (for the “0 h” and the “20 h No Treatment” groups), or UW Solution to which crystalline PrC-210, to achieve 30 mM [17], had been added, dissolved immediately, and then pH adjusted to the starting UW Solution pH of 7.4 by adding 0.0619 µL 5N NaOH per µmol of PrC-210 HCL salt (FW: 220). The half-life of PrC-210 thiol (active form) is approximately 3.5 h in physiologic pH solutions such as UW Solution and human blood [14]. Following in situ perfusion, the left BN kidney was surgically removed and then sutured by blunt anastomosis of vessels and ureter into the vacated left kidney site of the LEW recipient rat. The right LEW kidney was ligated and removed immediately before. Five minutes after surgical closure of the LEW rat, the rat received a systemic PrC-210 dose (121 ug PrC-210 HCl per gm body weight, which equals 0.24 X Maximum Tolerated Dose) by intraperitoneal injection. As shown in the Figure 1 schematic, the rat also received intraperitoneal injection doses of PrC-210 (0.24 MTD) at +4 h and +8 h following the transplant. Rats were euthanized at +20 h following transplant, and kidneys and plasma samples were collected for analysis. There were a minimum of five rats in each treatment group.

### 2.4. Serum BUN and Creatinine Measurements

BUN and creatinine were measured in serum samples using the Catalyst One Analyzer Technology (IDEXX Laboratories, Westbrook, ME, USA).

### 2.5. Enzyme-Linked Immunosorbent Assays

Assays were performed as described in the ELISA kit protocols (Rat TIMP-1, Cat# RTM-100; Rat MIP3-A, Cat# DY540; Rat TNF-alpha, Cat# RTA00; R&D Systems, Minneapolis, MN, USA). Briefly, dilutions of rat plasma were added to precoated plates, incubated for 2 h at 37 °C. Biotin-conjugated antibody specific for the assayed protein was then added and incubated for 1 h at 37 °C, washed, avidin-conjugated horseradish peroxidase was added, followed by washing and TMB substrate addition. The reaction was incubated for 10–30 min, stopped with sulfuric acid and read at 450 nm.

### 2.6. Proteome Profiler Rat Cytokine Array

Assays were performed essentially as described in the product protocol. Briefly, plasma was incubated with nitrocellulose membranes spotted with capture and control antibodies. After incubation, the membranes were washed and incubated with streptavidin HRP. In a deviation from protocol, we utilized SuperSignal West Femto (ThermoFisher, Madison, WI, USA; Cat# 34094) for chemiluminescent detection, as it gave a stronger signal. Blots were visualized on a FotoDyne gel doc system.

### 2.7. Histology

Formalin-fixed (10% formalin), paraffin-embedded, kidneys were cut into 5 um sections. Slides were deparaffinized, rehydrated from xylene through a graded ethanol series to water and subsequently treated as described below. Slides were scanned using a 20× objective in an Aperio Digital Pathology Slide Scanner. All H&E slides were reviewed by Dr. Weixiong Zhong, MD, PhD, transplant pathologist, and scored for ptc, glomerulitis (g), vasculitis (v)/intimal arteritis, interstitial inflammation (i) and C4d staining, according to Banff 2009 [21].

Separately, slides were assigned a blinded number, and non-overlapping digital images of renal tubules were taken at the interface between the medulla and the cortex from each H/E slide. Care was taken to not include large vessel lumens and glomeruli. Automated quantification of red and blue pixels in each 10× kidney image was performed using a custom macro written in ImageJ software (https://imagej.nih.gov/ij/index.html accessed on 13 April 2021). Red pixels reflected proximal tubular thickness including brush border. Nuclei were quantified in the blue channel. The ratio of blue nuclear pixels to red tubules provided an Inflammatory Infiltration Score for the white blood cell infiltration in the post-transplant kidneys. Scores were averaged and plotted using Graphpad Prism.

### 2.8. Activated Caspase Enzyme Activity

Activated caspase 3 and 7 activity in kidney homogenate supernates was determined using the Apo-ONE fluorescent substrate (Promega, Madison, WI, USA) [16]. Briefly, thawed kidneys were mixed with an 8-fold excess of lysis buffer containing 50 mM Na HEPES, pH 7.4, 100 mM NaCl, 1 mM EDTA, 10 mM DTT, 10% glycerol and homogenized at 4 °C for 30 s with an Omni tissue homogenizer. The kidney homogenate was centrifuged at 4 °C (16,000× *g*) in an Eppendorf microfuge for 20 min. The supernates were immediately assayed for caspase activity, and protein content by the Bradford method using bovine serum albumin as the standard. The activated caspase assay was performed as follows: 5 µL supernate (~40 μg of supernate protein) was diluted to a total volume of 50 µL with the above lysis buffer, was mixed with 50 μL of the undiluted Apo-ONE substrate in the well of a black, opaque, 96 well plate to initiate the 60 min reaction. Plates were shaken at 200 RPM at 37 °C for 60 min. The DEVD caspase substrate peptide cleavage was measured using a BMG Clariostar fluorescent plate reader at an excitation wavelength of 499 nm and an emission wavelength of 521 nm. A caspase standard was included in each experiment.

### 2.9. Rat Kidney Mitochondria

The purified mitochondrial fraction was prepared from homogenized rat kidneys by a standard centrifugation technique [22]. The purified mitochondria were suspended in 0.15 M Tris HCl buffer, pH 7.4.

To determine whether the addition of exogenous PrC-210 suppresses ROS-induced fragmentation of mitochondrial DNA [22], in a 25 µL reaction volume (in a PCR tube), we added: 10 µL purified mitochondria, 5 µL PrC-210 dilution or water (PrC-210 was added 10 min before the Fe^++^ + ADP +H_2_O_2_ •OH generator), and 10 µL containing FeCl_2_ (2.5 mM; FW:127), adenosine 5′-diphosphate sodium salt (10 mM; FW: 427) and H_2_O_2_ (0.003% final concentration). After 20 min at 37 °C, 10 µL of the reaction was mixed with 5 µL of 6× gel loading dye containing 0.3% SDS; tubes sat in 60 °C water for 1 min, 10 ul was then loaded into a well of a 1% agarose TAE gel, and after 60 min at 60 volts, gels were stained and photographed. A minimum of three replicates were done for each assay point to enable statistical comparison.

### 2.10. Statistical Analysis

Data are expressed as the means +/− STDs. Student’s t-tests were used to determine statistical difference and *p* values using GraphPad Prism 7.03 software. *p*-values less than 0.05 were considered significant.

## 3. Results

### 3.1. PrC-210 Suppression of Kidney Allograft Pathology

**Figure 2:**
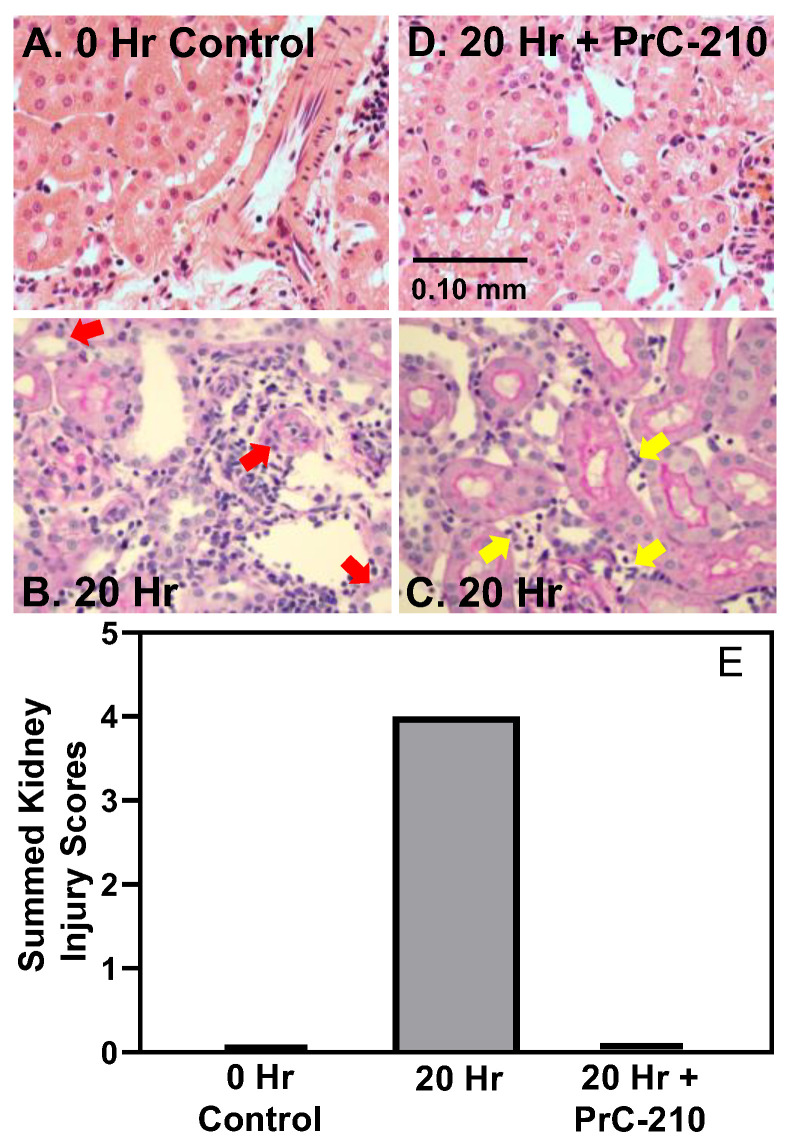
Histology of (**A**) BN kidney upon removal from BN rat. (**B**–**D**) BN kidneys 20 h following transplant into LEW rats. The “20 h” control kidneys (**B**,**C**) were flushed with room temperature UW Solution prior to transplant, and +PrC-210 kidneys (**D**) were flushed with room temperature UW Solution containing 30 mM PrC-210 prior to transplant, and recipient LEW rat also received three intraperitoneal systemic PrC-210 doses in the eight hours following transplant. Panel (**B**) red arrows highlight tubulitis pathology, and Panel C yellow arrows highlight peritubular capillaritis pathology. Panel (**E**) shows summed tubulitis and peritubular capillaritis severity scores for three kidneys in each of the indicated groups.

At 20 h post-transplant, the histology of the transplanted BN kidneys (Figure 2A–D) treated with UW Solution alone clearly showed increased Banff scores for tubulitis and peritubular capillaritis (red and yellow arrows); the summed pathology scores are shown in panel E. BN kidneys perfused with PrC-210-containing UW Solution and receiving post-implant intraperitoneal systemic doses of PrC-210 (Figure 2D) showed clearly suppressed inflammatory pathology to the kidney and suppressed Banff scores for tubulitis and peritubular capillaritis equal to Banff scores of the “0 h” control BN group (Figure 2E).

**Figure 3:**
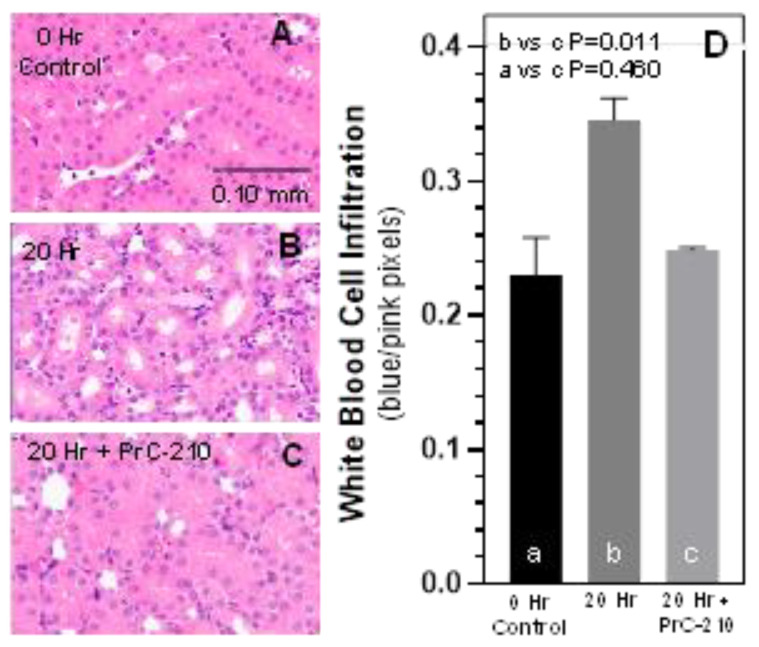
(**A**–**C**) Kidney histology 20 h following transplant. Operator randomly collected sample images of renal tubules from each of the indicated kidney groups in panels (**A**–**C**). Then, using an ImageJ macro (see Methods), H/E images (e.g., Figure 3 panels (**A**–**C**)) were analyzed, and above-threshold pink or blue pixels were selected and enumerated, and their ratio was then calculated and plotted to provide an estimate of kidney white blood cell infiltration (Panel (**D**)). In Panel (**D**), a,b,c designations are used to enable statistical comparisons between groups.

The “20 h” histology of the BN kidneys only flushed with UW Solution showed a significantly reduced thickness of the renal tubule brush border epithelium (Figure 3B) in comparison to the “0 h” control BN kidneys just removed from a BN rat (Figure 3A). The histology of BN kidneys receiving PrC-210 via both the perfusing UW Solution and intraperitoneal injections, clearly showed a preserved integrity of the renal tubular brush border (Figure 3C).

The same blinded histology sections were examined for mononuclear white cell infiltration. BN kidneys at 20 h only flushed with UW Solution showed a significant increase in the number of blue nuclei (scored as blue pixels), which reflects the infiltration of mononuclear white blood cells. In contrast, mononuclear infiltration in the BN kidneys flushed and treated with PrC-210 was significantly suppressed versus the untreated BN kidneys (*p* = 0.011) and the Inflammatory Infiltration Score was statistically the same as the 0 h control group (Figure 3D).

**Figure 4:**
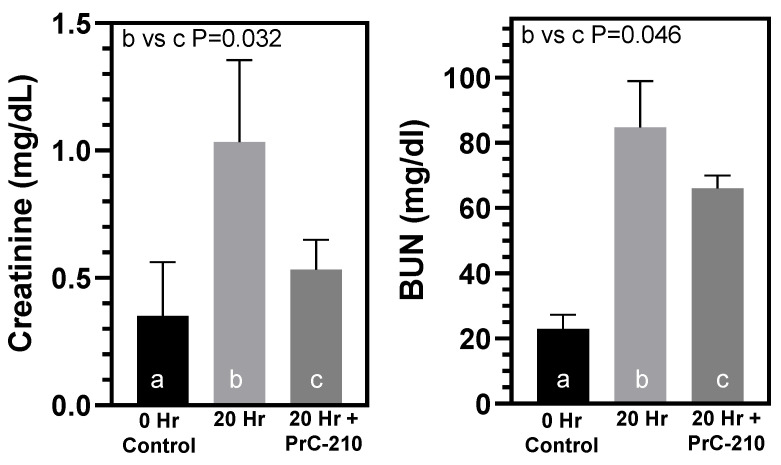
Effects of PrC-210 administration on kidney function 20 h after transplant of the BN kidney into a LEW rat. Rats either received no PrC-210 (“20 h”) or three IP PrC-210 injections in the 20 h period following transplant (“20 h + PrC-210”), before recipient rat was euthanized and serum was collected. Serum BUN and creatinine levels were determined as described in Methods. *p* values are indicated. a,b,c designations are used to enable statistical comparisons between groups.

Serum creatinine and serum BUN were also measured to assess function in the BN allograft kidneys 20 h after transplant (Figure 4).

Administration of PrC-210 conferred significant reductions in both the creatine (*p* = 0.032) and BUN (*p* = 0.046) kidney damage markers.

### 3.2. Activated Caspase Levels in Post-Transplant Kidneys

**Figure 5:**
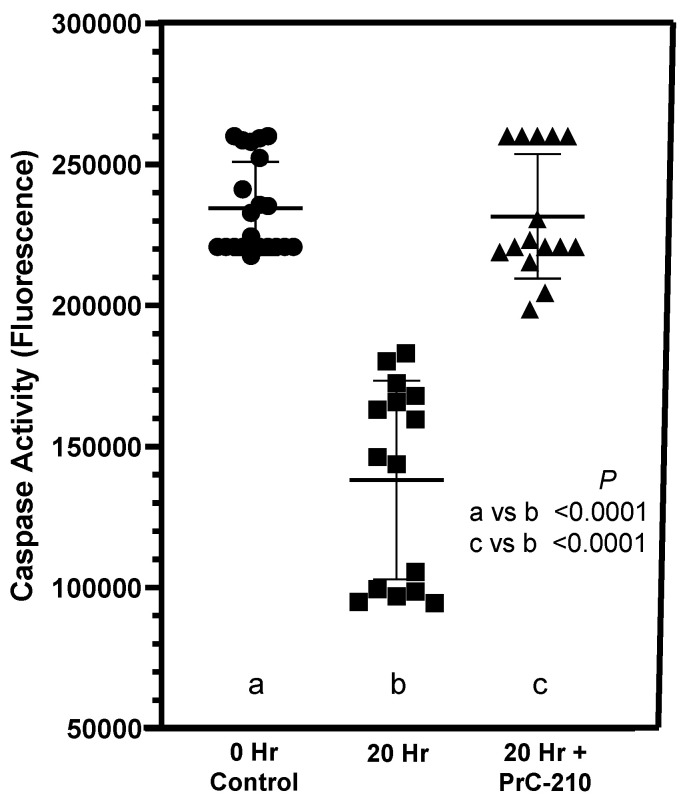
Caspase activity in post-transplant kidneys. Kidney supernatant activated caspase activity was measured enzymatically as described in Materials and Methods in a 60 min reaction. a,b,c designations are used to enable statistical comparisons between groups.

Levels of activated caspase in BN kidney homogenates were significantly reduced in BN allograft kidneys that were not exposed to PrC-210 treatment during the 20 h following transplant (Figure 5). Perfusion of BN kidneys with PrC-210-containing UW Solution and transplant into Lewis rats that received systemic PrC-210 doses resulted in the same activated caspase activity to that seen in the “0 h” control kidneys.

### 3.3. Inflammatory Cytokine Levels Following BN Kidney Allograft

**Figure 6:**
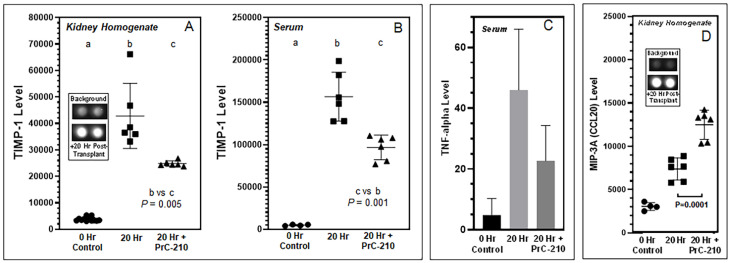
Effect of PrC-210 administration on serum and kidney homogenate cytokine levels 20 h after transplant of the BN kidney into a LEW rat. (**A**,**B**) TIMP-1, (**C**) TNF-alpha and (**D**) MIP-3A/CCL20 levels were determined by ELISA analysis. Serum collection and transplanted BN kidney harvest and homogenization were done as described in Methods. *p* values are indicated. a,b,c designations are used to enable statistical comparisons between groups.

In screening experiments, kidney homogenate supernates from 0 h controls and 20 h No Treatment kidneys were screened with the Proteome Profiler 29 cytokine array to detect altered, inflammation-associated, cytokine and chemokine expression levels 20 h post-transplant. As shown in the two Figure 6 microarray insets, changes were seen in TIMP-1, TNF-alpha and MIP-3a/CCL20. Individual ELISA plates were then used to quantify these changes in kidney homogenates and sera, now including rats treated with PrC-210 as well. Both TIMP-1 and TNF-alpha levels were increased 20 h post-transplant, and in both cases, their levels were decreased in the presence of PrC-210 (Figure 6A–C). MIP-3a/CCL20 levels were increased at 20 h, but they increased significantly higher in PrC-210-treated rats (Figure 6D).

### 3.4. PrC-210 Protection of Rat Kidney Mitochondria

Sustained mitochondrial function during and after kidney transplant is required for survival of the transplanted organ. ROS generated during transplant-associated ischemia, ischemia-reperfusion, and post-implant inflammation can all affect kidney mitochondrial performance and survival. Because of the important mitochondrial role, we isolated mitochondria from rat kidneys and determined whether PrC-210 at achievable pharmacologic concentrations could protect these organelles from an ROS insult.

**Figure 7:**
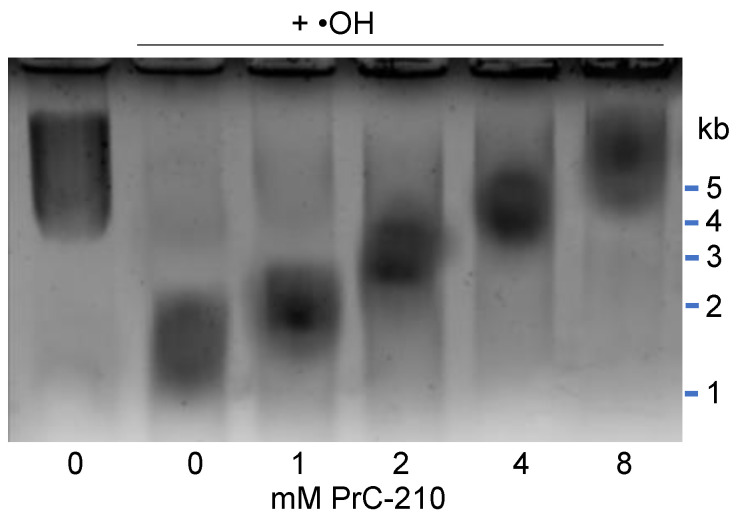
Suppression of hydroxyl radical-induced rat kidney mitochondrial DNA fragmentation by PrC-210 in a dose-dependent manner. Ten minutes before addition of the hydroxyl radical generator, PrC-210 was added to the incubations at the indicated concentrations. Following 20 min incubation at 37 °C, a reaction aliquot (10 µL) was mixed with 0.1% SDS loading dye and heated to 60 °C (1 min). Electrophoresis was performed, and gels were stained with ethidium bromide.

In Figure 7, purified rat kidney mitochondria incubated with an •OH generator [23]. Following the brief reaction, we saw significant ROS fragmentation of rat kidney mitochondrial DNA. Following the ROS insult, an aliquot of the mitochondria was solubilized in SDS-containing gel-loading buffer, and mitochondrial DNA was separated and “sized” using agarose gel chromatography (Figure 7). The ROS insult clearly reduced the mean size of the rat kidney mitochondrial DNA (lane b), and addition of PrC-210 to the mitochondria prevented the DNA breakage (lanes c–g) in a PrC-210 concentration-dependent manner.

## 4. Discussion

Allograft kidney transplantation, which triggers innate host cellular- and antibody-mediated rejection of the kidney, is a major contributor to short and long-term kidney damage during transplant, and the associated Delayed Graft Function seen in up to 50% of transplanted kidneys. We undertook this study to determine whether PrC-210 would be effective in suppressing the severity of the damage induced following allograft kidney transplant in a rat model that largely eliminates transplant ischemic time and its associated oxidative stress. Our assumption was that this approach should allow us to see the impact of PrC-210 on the post-transplantation inflammation insult with minimal ischemia interference.

The increase in TNF-alpha and substantial mononuclear infiltration demonstrate that allograft kidney transplantation induces pronounced acute inflammation in the 20 h after transplantation, and this was correlated with the damage of the kidney tubular cells seen in the kidney histology (Banff Scores). TNF-alpha is mainly produced by activated macrophages and is a cell signaling protein involved in acute inflammation. It is closely associated with the pathogenesis of acute and chronic allograft rejection [24].

In contrast to the above findings in non-treated BN kidneys, PrC-210 given as part of the UW Solution and administered systemically in the post-transplant rats reduced both TNF-alpha level and kidney infiltration by mononuclear cells, which are both signs of reduced acute inflammation. PrC-210 reduced the kidney damage as seen in the histological kidney injury scores (Banff Scores) to untreated background levels and lowered levels of both kidney pathology functional scores, creatinine and BUN.

Inflammation in untreated BN kidneys was associated with an increase in both TIMP-1 and MIP-3A/CCL20. By comparison, we saw that PrC-210 treatment significantly reduced the TIMP-1 level and significantly increased the MIP-3A/CCL20 level.

Tissue inhibitor of metalloproteinase-1 (TIMP-1) is an important regulator of extracellular matrix (ECM) synthesis and degradation. Excess ECM accumulation is the main pathological mechanism of fibrosis development during and after acute kidney injury. There is essentially no expression of TIMP-1 in normal kidney tissue [25], an observation which is corroborated in our Figure 6A,B, but TIMP-1 is known to be expressed in injured kidneys, mainly in renal tubular epithelial cells, renal tubular basement membrane and the cytoplasm of interstitial cells. Increased TIMP-1 expression was positively correlated with the simultaneous deterioration of renal function [26]. Rats treated with PrC-210 showed a profound reduction in TIMP-1 levels (*p* = 0.001), both in kidney homogenate and plasma; this implies that PrC-210 exerts a strong protective effect against transplantation-induced reorganization of the kidney extracellular matrix.

The chemokine MIP-3a/CCL20 activates the CCR6 receptor, which is expressed especially on regulatory T-cells (Tregs). CCL20 is expressed by tubular endothelial and interstitial cells and is also upregulated in kidneys with acute kidney injury. The CCL20–CCR6 pathway plays a vital role in Treg-mediated T-cell recruitment to the kidney, and Tregs have been described to have a positive role in kidney repair, transplant tolerance, and kidney survival. Both antibody blocking of the CCL20–CCR6 pathway, as well as the use of CCR6-deficient mice in acute kidney injury experiments, were shown to increase the severity of kidney failure and mortality [27]. This suggests, that clinically, CCL20–CCR6 pathway enhancement and Treg activation may be a possible therapeutic route to limit acute and chronic kidney injury [28]. In our study (Figure 6D), the MIP-3a/CCL20 level was significantly higher in PrC-210-treated rats than in untreated rats. We speculate that this is one of the reasons for both the (i) significantly lower recruitment of mononuclear cells to kidneys (Figure 3C) and (ii) the significantly reduced kidney damage (Figure 2) in the PrC-210-treated rats.

Normal kidney mitochondrial function, and importantly, insults to it during the kidney storage, implant, and post-implant inflammation steps are significant determinants of ROS injury, and kidney failure during transplant. It was thus significant that PrC-210 was shown to confer complete suppression of mitochondrial DNA fragmentation (Figure 7) at concentrations (2–4 mM) that have been achieved in the plasma of both mice and rats that were given either intraperitoneal or oral systemic 0.5 MTD doses of PrC-210 that were tolerated with no detectable toxicities [29].

**Figure 8:**
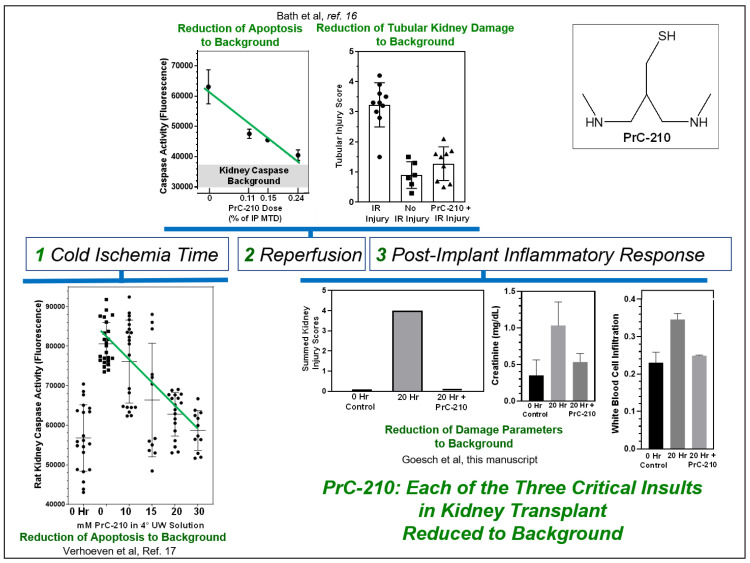
Schematic summarizing PrC-210 efficacy in suppressing each of the three organ insults that occur during kidney transplantation.

In our earlier kidney transplant-related studies [16,17], we saw substantial increases in activated caspase in kidneys exposed to “cold ischemia” and “ischemia-reperfusion” injury. These ischemia-induced insults to the kidneys were reduced to background by treatment with PrC-210 (Figure 8). In the studies of this manuscript (Figure 5), in which cold ischemia and ischemia-reperfusion were essentially eliminated by immediate transplant, there was no increase in activated caspase in transplanted kidneys. Rather, activated caspase was significantly reduced at +20 h in “No Drug Treatment” controls, and PrC-210 treatment completely eliminated this caspase reduction in +20 h rats and kept the caspase level stable. Our interpretation of these interesting results is that absent any significant ischemia-induced free radical insult through ROS and RNS to the post-transplant kidneys, there is no associated cell death and apoptosis markers like activated caspases. Rather, in these allograft kidney transplants, inflammatory signals from newly expressed cyto- and chemokines now regulate cell metabolism, which includes influencing the apoptosis pathway. The literature describes that overexpression of TIMP-1 leads to suppression of apoptosis [26]. Our caspase results (Figure 5) support this described TIMP-1 effect, and they imply that TIMP-1 is important in regulating the pathophysiology of cell damage after kidney allograft transplantation. In corroboration of the earlier PrC-210 suppression of TIMP-1 expression (Figure 6A,B), PrC-210 treatment completely ablated the caspase change, keeping the caspase levels at the same level seen in the control “0 h” kidneys. Because the reduced PrC-210 serum TIMP-1 levels at +20 h (Figure 6B) accurately reflect the significant suppression of allograft: (i) apoptosis (Figure 5), (ii) histologic pathology (Figure 2 and Figure 3) and (iii) inflammatory cell infiltration (Figure 3), we expect that monitoring serum TIMP-1 levels in human kidney allograft recipients will be a logical way to monitor PrC-210 clinical efficacy in future clinical trials.

In our work to date [16,17], we have shown that PrC-210 is able to protect transplanted kidneys against both the cold-ischemia and ischemia-reperfusion insults. In this study, we now see that PrC-210 also protects allograft kidneys from the non-ischemia inflammatory insults that occur after kidney implant. PrC-210 significantly reduces levels of acute inflammatory cytokines, such as TNF-alpha, and suppresses expression of the TIMP-1 chemokine. Both of these events, and potentially, further supported by additional CCL20 expression, would be expected to: (i) reduce allograft kidney damage, (ii) suppress T-cell recruitment to the kidney, and (iii) suppress activation of the innate and adaptive immune system. In Figure 8, we summarize these findings to support the role that we feel PrC-210 can play in human kidney transplantation; it suppresses: (i) cold ischemia reactive oxygen species (ROS) and reactive nitrogen species (RNS) damage to background [17], (ii) ischemia-reperfusion ROS damage to background [16], and (iii) allograft inflammation damage substantially, in some cases, to background.

Since the primary PrC-210 mechanism of action for PrC-210 is scavenging oxygen and nitrogen free radicals, this implies that these free radicals are an important contributor to the kidney damage seen in non-ischemic conditions, i.e., the allograft-associated inflammation studied in this manuscript.

In summary, this suggests that PrC-210 could provide broadly applicable organ protection for many allograft transplantation conditions; it could protect transplanted kidneys during and after all stages of the transplantation process—from organ donation, through transportation, re-implantation and the post-operative inflammation—to minimize acute and chronic rejection.

## Data Availability

The study data are fully available within this manuscript.

## References

[1] Wang H., Naghavi M., Allen C., Barber R.M., Bhutta Z.A., Casey D.C., Charlson F.J., Chen A.Z., Coates M.M., Coggeshall M. (2016). Global, regional, and national life expectancy, all-cause mortality, and cause-specific mortality for 249 causes of death, 1980–2015: A systematic analysis for the Global Burden of Disease Study 2015. Lancet.

[2] Treat E., Chow E., Peipert J.D., Waterman A., Kwan L., Massie A.B., Thomas A.G., Bowring M.G., Leeser D., Flechner S. (2017). Shipping living donor kidneys and transplant recipient outcomes. Arab. Archaeol. Epigr..

[3] Weight S.C., Bell P.R., Nicholson M.L. (1996). Renal ischaemia-reperfusion injury. Br. J. Surg..

[4] Sellarés J., De Freitas D.G., Mengel M., Reeve J., Einecke G., Sis B., Hidalgo L.G., Famulski K., Matas A., Halloran P.F. (2012). Understanding the Causes of Kidney Transplant Failure: The Dominant Role of Antibody-Mediated Rejection and Nonadherence. Am. J. Transplant..

[5] Siedlecki A., Irish W., Brennan D.C. (2011). Delayed Graft Function in the Kidney Transplant. Arab. Archaeol. Epigr..

[6] Hidalgo L.G., Campbell P.M., Sis B., Einecke G., Mengel M., Chang J., Sellares J., Reeve J., Halloran P.F., Hidalgo L.G. (2009). De NovoDonor-Specific Antibody at the Time of Kidney Transplant Biopsy Associates with Microvascular Pathology and Late Graft Failure. Arab. Archaeol. Epigr..

[7] Loupy A., Hill G.S., Jordan S.C. (2012). The impact of donor-specific anti-HLA antibodies on late kidney allograft failure. Nat. Rev. Nephrol..

[8] Huang G., Wilson N.A., Reese S.R., Jacobson L.M., Zhong W., Djamali A. (2014). Characterization of Transfusion-Elicited Acute Antibody-Mediated Rejection in a Rat Model of Kidney Transplantation. Arab. Archaeol. Epigr..

[9] Jordan S.C., Pescovitz M.D. (2006). Presensitization: The Problem and Its Management. Clin. J. Am. Soc. Nephrol..

[10] Stegall M., Gloor J., Winters J., Moore S., DeGoey S. (2006). A Comparison of Plasmapheresis Versus High-Dose IVIG Desensitization in Renal Allograft Recipients with High Levels of Donor Specific Alloantibody. Arab. Archaeol. Epigr..

[11] Peebles D.D., Soref C.M., Fahl W.E. (2012). ROS-scavenger and radioprotective efficacy of the new PrC-210 aminothiol. Radiat. Res..

[12] Soref C.M., Hacker T.A., Fahl W.E. (2012). A New Orally Active, Aminothiol Radioprotector-Free of Nausea and Hypotension Side Effects at Its Highest Radioprotective Doses. Int. J. Radiat. Oncol..

[13] Techapiesancharoenkij N., Fiala J.L., Navasumrit P., Croy R.G., Wogan G.N., Groopman J.D. (2015). Sulforaphane, a cancer chemopreventive agent, induces pathways associated with membrane biosynthesis in response to tissue damage by af-latoxin B1. Toxicol. Appl. Pharmacol..

[14] Jermusek F., Benedict C., Dreischmeier E., Brand M., Uder M., Jeffery J.J., Ranallo F.N., Fahl W.E. (2018). Significant Suppression of CT Radiation-Induced DNA Damage in Normal Human Cells by the PrC-210 Radioprotector. Radiat. Res..

[15] Hacker T.A., Diarra G., Fahl B.L., Back S., Kaufmann E., Fahl W.E. (2019). Significant reduction of ischemia-reperfusion cell death in mouse myocardial infarcts using the immediate-acting PrC-210 ROS-scavenger. Pharmacol. Res. Perspect..

[16] Bath N.M., Fahl W.E., Redfield R.R. (2019). Significant reduction of murine renal ischemia-reperfusion cell death using the im-mediate-acting PrC-210 reactive oxygen species-scavenger. Transplant. Direct..

[17] Verhoven B.M., Karim A.S., Bath N.M., Fahl C.J.S., Wilson N.A., Redfield R.R., Fahl W.E. (2020). Significant Improvement in Rat Kidney Cold Storage Using UW Organ Preservation Solution Supplemented with the Immediate-Acting PrC-210 Free Radical Scavenger. Transplant. Direct.

[18] Giese A.P.J., Guarnaschelli J.G., Ward J.A., Choo D.I., Riazuddin S., Ahmed Z.M. (2015). Radioprotective Effect of Aminothiol PrC-210 on Irradiated Inner Ear of Guinea Pig. PLoS ONE.

[19] Copp R.R., Peebles D.D., Fahl W.E. (2011). Synthesis and growth regulatory activity of a prototype member of a new family of aminothiol radioprotectors. Bioorganic Med. Chem. Lett..

[20] Fahl W.E., Peebles D., Copp R.R. (2004). Amino Thiol Compounds and Compositions for Use in Conjunction with Cancer Therapy. U.S. Patent.

[21] Haas M., Sis B., Racusen L.C., Solez K., Glotz D., Colvin R.B., Castro M.C.R., David D.S.R., Davidneto E., Bagnasco S.M. (2014). Banff 2013 Meeting Report: Inclusion of C4d-Negative Antibody-Mediated Rejection and Antibody-Associated Arterial Lesions. Arab. Archaeol. Epigr..

[22] Hruszkewycz A.M. (1988). Evidence for mitochondrial DNA damage by lipid peroxidation. Biochem. Biophys. Res. Commun..

[23] Floyd R.A., Watson J.J., Wong P.K. (1984). Sensitive assay of hydroxyl free radical formation utilizing high pressure liquid chromatography with electrochemical detection of phenol and salicylate hydroxylation products. J. Biochem. Biophys. Methods.

[24] Sang-Kyu P. (2016). Association between tumor necrosis factor-alpha (TNF-α) polymorphism (−308, G/A) and acute rejec-tion of solid organ allograft: A meta-analysis. Int. J. Clin. Exp. Med..

[25] Qiang Y. (2012). Expression of MMP-2 and TIMP-1 in renal tissue of patients with chronic active antibody-mediated renal graft rejection. Diag. Pathol..

[26] Gangyong L. (1999). Tissue Inhibitor of Metalloproteinase-1 inhibits apoptosis of human breast epithelial cells. Cancer Res..

[27] González-Guerrero C., Morgado-Pascual J.L., Cannata-Ortiz P., Ramos-Barron M.A., Gómez-Alamillo C., Arias M., Mezzano S., Egido J., Ruiz-Ortega M., Ortiz A. (2018). CCL20 blockade increases the severity of nephrotoxic folic acid-induced acute kidney injury. J. Pathol..

[28] Min H. (2016). Regulatory T cells in kidney disease. and transplantation. Kidney Int. J..

[29] Dreischmeier E., Fahl W.E. (2021). Determination of Plasma Levels of the Active Thiol Form of the Direct-Acting PrC-210 ROS-Scavenger Using a Fluorescence-Based Assay. Anal. Biochem..

